# Coronary Arteriovenous Fistulas in Adult Patients: Surgical Management and Outcomes

**DOI:** 10.21470/1678-9741-2017-0005

**Published:** 2017

**Authors:** Sebnem Albeyoglu, Mustafa Aldag, Ufuk Ciloglu, Murat Sargin, Tugba Kemaloglu Oz, Hakan Kutlu, Sabri Dagsali

**Affiliations:** 1 Siyami Ersek Thoracic and Cardiovascular Surgery Training and Research Hospital, Istanbul, Turkey.

**Keywords:** Coronary Vessels, Arteriovenous Fistula/Surgery, Outcome Assessment (Health Care)

## Abstract

**Objective:**

The aim of this study was to describe the demographic, clinical and anatomic characteristics of coronary arteriovenous fistulas in adult patients who underwent open cardiac surgery and to review surgical management and outcomes.

**Methods:**

Twenty-one adult patients (12 female, 9 male; mean age: 56.1±7.9 years) who underwent surgical treatment for coronary arteriovenous fistulas were retrospectively included in this study. Coronary angiography, chest X-ray, electrocardiography and transthoracic echocardiography were preoperatively performed in all patients. Demographic and clinical data were also collected. Postoperative courses of all patients were monitored and postoperative complications were noted.

**Results:**

A total of 25 coronary arteriovenous fistulas were detected in 21 patients; the fistulas originated mainly from left anterior descending artery (n=9, 42.8%). Four (19.4%) patients had bilateral fistulas originating from both left anterior descending and right coronary artery. The main drainage site of coronary arteriovenous fistulas was the pulmonary artery (n=18, 85.7%). Twelve (57.1%) patients had isolated coronary arteriovenous fistulas and 4 (19.4%), concomitant coronary artery disease. Twenty (95.3%) of all patients were symptomatic. Seventeen patients were operated on with and 4 without cardiopulmonary bypass. There was no mortality. Three patients had postoperative atrial fibrillation. One patient had pericardial effusion causing cardiac tamponade who underwent reoperation.

**Conclusion:**

The decision of surgical management should be made on the size and the anatomical location of coronary arteriovenous fistulas and concomitant cardiac comorbidities. Surgical closure with ligation of coronary arteriovenous fistulas can be performed easily with on-pump or off-pump coronary artery bypass grafting, even in asymptomatic patients to prevent fistula related complications with very low risk of mortality and morbidity.

**Table:** 

Abbreviations, acronyms & symbols	
ASD CABG CAVF CPB ECG LAD LIMA LVEF RCA	= Atrial septal defect = Coronary artery bypass grafting = Coronary arteriovenous fistula = Cardiopulmonary bypass = Electrocardiography = Left anterior descending artery = Left internal mammary artery = Left ventricular ejection fraction = Right coronary artery

## INTRODUCTION

Coronary arteriovenous fistulas (CAVFs) are uncommon in the adult population, and can be defined as a condition where the coronary blood flow is usually shunted into a cardiac chamber, great vessels, or other structures, bypassing the myocardial capillary network resulting in a coronary steal phenomenon with myocardial ischemia, causing morbidity and mortality^[1,2]^. CAVFs are commonly congenital, but rarely may be acquired due to angioplasty, postcardiac surgery or after trauma^[3,4]^. CAVFs are present in 0.002% of the general population and they are detected in 0.3-0.8% of the patients undergoing diagnostic cardiac catheterization^[5,6]^. Although some previous studies reported frequent origin of CAVFs from the right coronary artery (RCA), some authors mentioned that CAVFs originate mostly from left anterior descending coronary artery (LAD)^[7,8]^. Generally patients with CAVFs remain asymptomatic but due to increasing age and shunt ratio they can become symptomatic. Symptoms include dyspnea on exertion, fatigue, angina pectoris and occasionally complications of congestive heart failure, myocardial infarction, pericardial or pleural effusion, cardiac arrhythmias and rupture of dilated aneurysmal coronary arteries^[8-10]^. Although the incidence, angiographic/anatomical characteristics, natural history and pathologic behavior of the entity are well established, only limited information is available regarding the surgical management and outcomes in adult patients. Also, studies on CAVFs of adults are few in comparison with pediatric age studies, majority of them being case reports.

The aim of this study is to present our 10 years’ experience in CAVFs surgery, enlighten its clinical features and association with other cardiac diseases as well as the challenges faced in surgical management and outcomes. The current study included 21 adult patients with 7 different anatomical locations of CAVFs treated by various surgical procedures. Surgical treatment strategies and postoperative outcomes were reviewed.

## METHODS

Twenty-one patients (12 female, 9 male) with CAVFs operated between 2005 and 2015 in our institution were included in this retrospective study. Coronary angiography was performed in all patients due to their presenting symptoms and other concomitant cardiac disorders. Chest X-ray, twelve-lead electrocardiography (ECG) and transthoracic echocardiography were performed for all patients. Preoperative demographic data and clinical symptoms, including gender, left ventricular ejection fraction (LVEF), pulmonary hypertension, main complaints, history of occlusive coronary artery disease or myocardial infarction, hypertension, diabetes and extra-cardiac arteriopathy were also noted. To further define anatomical characteristics of the cases, the origin and drainage sites of each fistula were assessed by both an invasive cardiologist and a cardiac surgeon. Demographic and clinical parameters of patients are listed in Table 1.

**Table 1:** Demographical and clinical parameters of patients.

Parameters		Min-Max	Mean±SD
Age (year)		34-69	56.11±7.9
LVEF (%)		35-70	56.19±9.21
		**n**	**%**
Gender	Female	12	57.14
	Male	9	42.85
Associated Pathologies	Atrial septal defect	1	4.7
	Coronary artery disease	4	19.04
	Aortic valve stenosis	1	4.7
	Mitral regurgitation	4	19.04
	Tricuspid regurgitation	1	4.7
	Isolated CAVF	12	57.1
Risk Factors	Arterial hypertension	7	33.3
	Diabetes on insulin	6	27.2
	Extracardiac arteriopathy	1	4.7
	Myocardial ischemia	3	14.2
	Pulmonary hypertension	1	4.7

CAVFs=coronary arteriovenous fistulas; LVEF=left ventricular ejection fraction

All operations were performed via sternotomy using cardiopulmonary bypass (CPB) or off-pump beating heart technique. The indications for using CPB were coexisting atrial septal defect (ASD), valve or coronary occlusive disease and unfavorable anatomy of fistulas in this study. Postoperative course of all patients was monitored and postoperative complications were noted. Patients who had any symptoms or cardiac murmurs auscultated postoperatively were examined in detail. Coronary arteriography was performed to patients who had any evidence of myocardial ischemia (presence of angina, elevation of cardiac markers or ischemia in myocardial perfusion scintigraphy) and any residual shunting was investigated.

### Ethics Statement

This retrospective study was approved by the HNEAH-KAEK Clinical Research Ethics Committee (Number: HNEAH-KAEK 2016/KK/29).

## RESULTS

**Fig.1:**
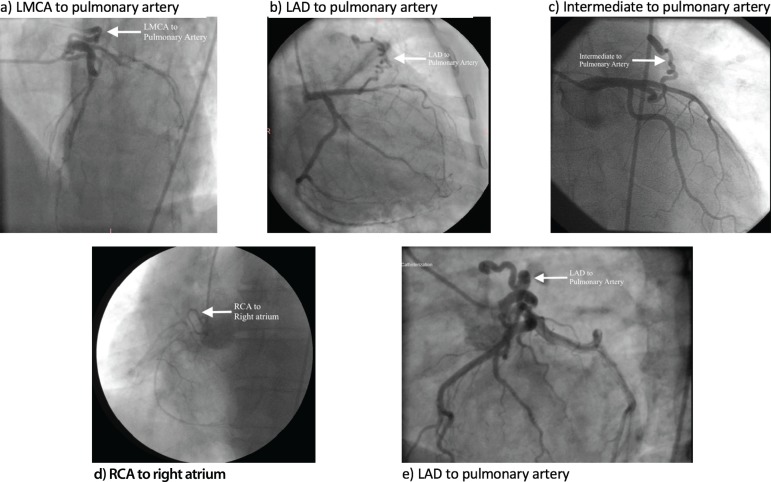
Angiographic images of coronary artery arteriovenous fistulas.

In the last decade, 21 adult patients with CAVFs were operated on in our institution. Twelve patients were female and nine male. The mean age was 56.1±7.9 years (range: 34-69 years). A total of 25 CAVFs were detected in the entire cohort; CAVFs originated mainly from LAD (n=9, 42.8%), the rest taking origin from diagonal artery (n=3, 14.2%), circumflex artery (n=2, 9.5%), right coronary artery (RCA) (n=2, 9.5%), and left main stem (n=1, 4.7). Four (19.4%) patients had bilateral fistulas originating from both LAD and RCA (Figure 1).

The main site of drainage of CAVFs were the pulmonary artery (n=18, 85.7%). The other sites were the right atrium, superior vena cava and the coronary sinus. The anatomical features of CAVFs are presented in Table 2. Twelve (57.1%) patients had isolated CAVFs, four (19.4%) had concomitant coronary artery occlusive disease, one (4.7%) had atrial septal defect (ASD), four (19.4%) had mitral valve regurgitation, one (4.7%) had tricuspid valve regurgitation and one patient had (4.7%) aortic valve stenosis.

**Table 2:** Anatomical features of CAVFs.

		n	%
**Origin**	Left main stem	1	4.7
	Left anterior descending artery	9	42.8
	Diagonal artery	3	14.2
	Left circumflex artery	2	9.5
	Right coronary artery	2	9.5
	Both left and right coronary	4	19.04
**Drainage**	Right atrium	1	4.7
	Pulmonary artery	18	85.7
	Coronary sinus	1	4.7
	Superior vena cava	1	4.7

CAVFs=coronary arteriovenous fistulas

Only one among all patients was asymptomatic (n=1, 4.7%) who was incidentally diagnosed upon routine physical examination and echocardiography, while all others were symptomatic.

Main sign was continuous murmur and the main symptom was angina pectoris in isolated CAVFs patient group, but if the patients had any additional cardiac disorder, symptoms of the concomitant disease were more prominent. Even continuous murmur (n=18, 85.7%) was the main symptom in the isolated CAVFs patient group, syncope was confirmed in only one (n=1, 4.7%) patient, who had aortic valve stenosis. Angina pectoris was determined in patients with coronary disease and isolated CAVFs patients. The most common signs and symptoms (Table 3) included angina pectoris (n=12, 57.1%), dyspnea on exertion (n=9, 42.8%), respectively. Four patients within the ASD group, aortic valve stenosis and mitral valve regurgitation had cardiomegaly with a cardiothoracic ratio of higher than 0.6. Three patients had hepatomegaly. With the exception of two patients who had developed congestive heart failure, all remaining patients were New York Heart Association Functional class II or III.

**Table 3:** Symptoms and signs.

		n	%
Asymptomatic		1	4.7
Symptomatic	Angina pectoris	12	57.1
	Dyspnea on exertion	9	42.8
	Congestive heart failure	2	9.5
	Continuous murmur	18	85.7
	Cardiomegaly	4	19.04
	Hepatomegaly	3	14.2
	Syncope	1	4.7

Operation was performed in all patients via median sternotomy. Seventeen patients were operated under CPB with mild (28-32º C) hypothermia, topical cooling, and antegrade application of blood cardioplegia after clamping of the ascending aorta and of the coronary fistula near the drainage site. Four patients who had isolated CAVFs were operated without CPB. Fifteen (71.4%) patients underwent fistula closure with additional coronary artery bypass graft (CABG) surgery, three (14.2%) patients had only simple ligation of coronary artery fistulas both proximally and distally at the origin and the drainage site. Five (23.8%) patients had simultaneous valve surgery, two patients had valve surgery with additional CABG and one patient received concomitant closure of ASD. Three (14.2%) fistulas were treated by ligating their distal ends through the pulmonary arteriotomy. The operations performed in association with CAVF closure are listed in Table 4.

**Table 4:** Surgical procedures performed in the patients with CAVFs.

Patient nº	Origin	Drainage	Main Diagnosis	Operation
1	LAD	PA	Isolated	FC+CABG (Off-Pump)
2	Dia	PA	Aortic stenosis	FC+AVR
3	LAD	PA	Isolated	FC+CABG
4	RCA	Right atrium	Mitral regurgitation	FC+CABG+MRA
5	Dia	PA	Mitral regurgitation	FC+MVR
6	Cx	PA	Isolated	FC
7	Dia	PA	Mitral and Tricuspid regurgitation	FC+MVR+TDV
8	RCA	SVC	Isolated	FC
9	LAD	PA	Isolated	FC+CABG
10	LAD+RCA	PA	Isolated	FC+CABG (Off-Pump)
11	LMCA	PA	Coronary occlusive disease	FC+CABG
12	LAD+RCA	PA	Isolated	FC (Off-Pump)
13	LAD	PA	ASD and Mitral regurgitation	FC+ASD+MVR+CABG
14	LAD	PA	Coronary occlusive disease	FC+CABG
15	LAD	PA	Isolated	FC+CABG
16	LAD+RCA	PA	Isolated	FC+CABG
17	LAD	PA	Coronary occlusive disease	FC+CABG
18	LAD	PA	Isolated	FC+CABG
19	LAD+RCA	PA	Isolated	FC+CABG
20	LAD	PA	Coronary occlusive disease	FC+CABG (Off-Pump)
21	Cx	Coronary Sinus	Isolated	FC+CABG

ASD=atrial septal defect; AVR=aortic valve replacement; CABG=coronary artery bypass grafting; Cx=circumflex coronary artery; Dia: Diagonal coronary artery; FC=fistula closure; LAD=left anterior descending artery; MRA=mitral ring annuloplasty; MRV=mitral valve replacement; RCA=right coronary artery; TDV=tricuspid de Vega annuloplasty

There was no operative or hospital mortality in any of the patients. Three (14.2%) patients developed postoperative atrial fibrillation requiring antiarrhythmic medications. One (4.7%) patient had pericardial effusion causing cardiac tamponade and underwent reoperation. After a mean intensive care unit stay of 1.4±0.8 days and hospital stay of 7.6±3.1 days, all patients were discharged from the hospital. Three of patients (n=3, 14.2%) had angina pectoris postoperatively and underwent coronary angiography, any residual fistula was not observed. The patients were submitted to echocardiography during postoperative course which revealed no residual shunts in any of the patients.

## DISCUSSION

Coronary arteriovenous fistulas are very rare cardiac anomalies. They were first described by Krause^[11]^, in 1865, and the first surgical treatment was also performed by Bjork and Crafoord^[12]^, in 1947. CAVFs may be congenital or acquired; they are generally asymptomatic in younger patients but due to increased age and shunts, and due to concomitant cardiac disease CAVFs can become symptomatic. Symptoms include dyspnea on exertion, fatigue, angina pectoris. Sometimes patients may present with complications of congestive heart failure, myocardial infarction resulting from steal phenomenon, pericardial or pleural effusion, cardiac arrhythmias, endocarditis or rupture of dilated aneurysmal coronary arteries^[7-10]^. In this study, 20 (95.23%) patients were symptomatic due to presence of underlying cardiac disease. The most common symptoms were angina pectoris and dyspnea on exertion.

CAVFs may cause angina without presence of accompanying occlusive vessel lesions, because a significant amount of coronary blood flow is directed towards another chamber via the fistula. The resting coronary flow is continuously kept at a high rate in order to compensate for this stolen blood flow, hence when exercising, myocardial perfusion may be inadequate due to inability of coronary flow reserve to be augmented. Therefore, ischemic symptoms may occur when the fistula is accompanied by uncritical atherosclerotic stenosis especially during exercise.

**Fig. 2:**
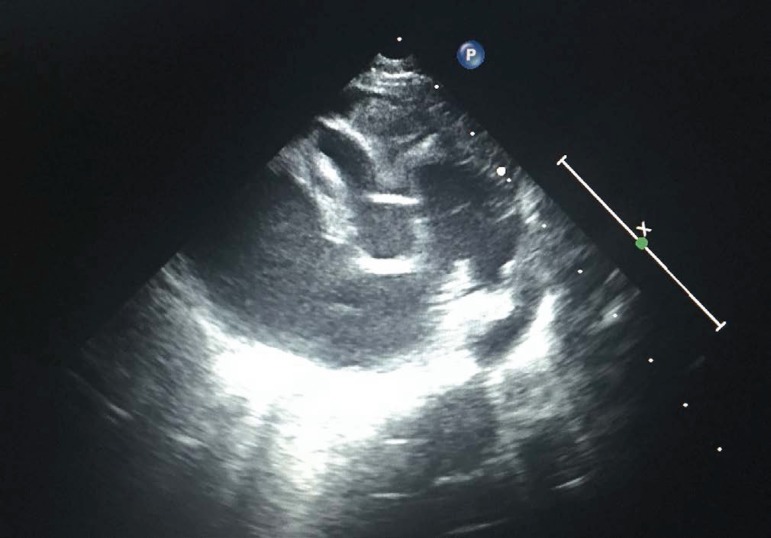
A transthoracic image of CAVF from the right coronary artery to right atrium.

The main diagnostic test for CAVFs is cardiac catheterization with coronary angiogram. Most fistulas are small and found incidentally during catheterization. However, detailed anatomy of fistulas cannot always be revealed by selective coronary angiogram. In such cases, computerized tomography coronary angiogram, transthoracic echocardiography combined with Doppler color flow imaging (Figure 2), and magnetic resonance imaging may provide further detail noninvasively^[1,8,13]^.

Most authors reported that CAVFs commonly originate from RCA (40%-50%) than other coronary arteries^[1,9,10,14-16]^, however, in our study we found 42.8% of CAVFs to arise from LAD (most frequently) and 14.8% from diagonal coronary artery. Recently, results similar to our study have been reported by authors^[7,8,17,18]^. Drainage of CAVFs into the left heart chambers is very rare^[3]^, and we had no such case in our series. Fistulas draining into the pulmonary artery are the most common entity, especially among adult patients in the literature^[7,8,14,18]^. Pulmonary artery (85.7%) was the most common drainage site of CAVFs in our study.

Multiple fistulas may be present in 11% to 16% of patients, and fistulas might originate from two separate coronary arteries in 4% to 18% of the cases^[1,2]^. Four (19.04%) of our patients had double CAVFs originating both from the left coronary artery and RCA. Until now, only a few cases have been reported with CAVF from RCA or circumflex coronary artery to superior vena cava^[4,17]^. In our series, one patient had CAVFs from RCA to superior vena cava and one patient had CAVFs from circumflex coronary artery to coronary sinus.

Bernhardt et al.^[14]^ reported successful treatment of a dilated circumflex artery and coronary sinus fistula. Patient presented with chest pain during exertion. They ligated the circumflex artery both close to the main stem and to the coronary sinus. Also first marginal branch was revascularized with the left internal mammary artery (LIMA). We performed fistula closure with additional on-pump CABG using left internal mammary artery (LIMA) for treatment of the patient who had isolated CAVF from circumflex coronary artery to coronary sinus. In our series, 15 patients underwent concomitant CABG and LIMA graft was used in 11 patients. There was no surgical morbidity or mortality. In case of patients who require additional CABG, we recommend the use of LIMA grafts because of the best graft patency rates, especially in younger patients with normal life expectancy.

Currently, it is widely accepted that all symptomatic CAVF patients should be treated surgically. There still remains a dispute for the surgical treatment of asymptomatic patients. Our study showed that surgical treatment of CAVFs when coexisting with other cardiac diseases is a safe procedure with low mortality and morbidity in accordance with the literature^[3,7,8,10,14,19]^. Thus, operating asymptomatic adult patients with low mortality and morbidity may reduce or eliminate the risk of subsequent development of complications such as myocardial infarction. Also, Canga et al.^[20]^ reported that large fistulas originating from the proximal segments of coronary arteries may increase the likelihood of atherosclerosis and myocardial infarction even in asymptomatic patients with no evidence of ischemia in noninvasive tests or dilatation of cardiac chambers, and should therefore be closed.

Surgical management and operative planning should depend on concomitant cardiac comorbidities, the size and the anatomic location of CAVFs. Additional CABG should be performed in fistula closure surgery even if 1- surgical access to fistula is difficult and challenging, 2- co-existing occlusive coronary artery disease is present. Although surgical outcomes of CAVFs are satisfactory and surgery is the gold standard for treatment, catheter-based managements using different devices, occluders and coils may be acceptable alternatives to surgery because of their easy manipulation, good results with high closure rate, and low morbidity and mortality rates^[21,22]^. However, in the presence of large CAVFs with high flow shunts, multiple communications and terminations, aneurysmal formation and necessity of simultaneous coronary bypass or valve surgery, transcatheter closure of the fistulas could not be performed in these groups of patients. In case of isolated CAVFs with convenient anatomy, catheter-based managements using different devices, occluders and coils may be considered as alternative treatment methods, but surgery is the only indication like the entire cohort due to presence of concomitant diseases.

### Limitations of the Study

Our study has some limitations which should be kept in mind while interpreting the results. First, the number of patient included in the study was relatively low. The main reason for this is that coronary arteriovenous fistulas are very rare cardiac anomalies in adult population. The second limitation of this study is the long-term follow-up periods. We could not collect all patients current contact information due to retrospective study design, therefore long-term follow up results are not included in this study.

## CONCLUSION

In conclusion, the most common type of CAVFs in our study was from LAD to pulmonary artery, whereas RCA to any ventricle or pulmonary artery was considered the most common type of coronary fistulas in previous studies. Surgical closure with ligation of CAVFs can be performed easily on the outer surface of the heart with CPB or off-pump beating heart technique. In addition, we recommend closure of CAVFs in all cases with additional concomitant cardiac surgery, even in asymptomatic patients, to prevent fistula related complications with very low risk of mortality and morbidity.

**Table:** 

Authors’ roles & responsibilities	
SA	Conception and study design; realization of operations and/or trials; manuscript redaction or critical review of its content; final manuscript approval
MA	Conception and design study; analysis and/or data interpretation; statistical analysis; final manuscript approval
UC	Realization of operations and/or trials; manuscript redaction or critical review of its content; final manuscript approval
MS	Statistical analysis; manuscript redaction or critical review of its content; final manuscript approval
TKO	Analysis and/or data interpretation; statistical analysis; manuscript redaction or critical review of its content; final manuscript approval
HK	Realization of operations and/or trials; manuscript redaction or critical review of its content; final manuscript approval
SD	Manuscript redaction or critical review of its content; final manuscript approval
